# Correlation between presence of lower limb varicose veins and deep venous thrombosis

**DOI:** 10.1590/1677-5449.200081

**Published:** 2020-07-31

**Authors:** Daniel Guimarães Cacione, Frederico do Carmo Novaes, José Carlos Costa Baptista Silva

**Affiliations:** 1 Universidade Federal de São Paulo – UNIFESP, Escola Paulista de Medicina – EPM, São Paulo, SP, Brasil.

The association between presence of lower limb varicose veins and occurrence of deep venous thrombosis (DVT) is a controversial subject in the literature. This issue emerged from studies that attempted to determine the risk factors for DVT (primarily postoperative DVT) to guide use of DVT prophylaxis and, in parallel, from research into superficial venous thrombosis (SVT, also known as superficial thrombophlebitis), since it can course with, or may be linked to, DVT. The controversy arises because the studies that investigated this association were limited by numerous problems that preclude a reasonable degree of certainty. In theory, the most appropriate study design for investigation of the association between a risk factor (e.g., lower limb varicose veins) and a disease (e.g., DVT) is the cohort study. However, this study design has not been used to prove the association. A slightly lower degree of reliability is afforded by control studies, in which we start with patients who have the disease and search for risk factors for its development. This is the design used by the majority of studies into the subject.

Two case-control population studies investigated the association between varicose veins and DVT: Heit et al.^1^ and Müller-Bühl et al.,^2^ published in 2002 and 2012, respectively. The study by Heit et al.,^1^ investigated a total of 1,250 patients over a 25-year follow-up period, divided into two groups: 625 people with DVT and pulmonary embolism (PE) were compared with 625 people in a control group without DVT or PE. Using multivariate logistic analysis, they found an association between presence of varicose veins and development of DVT and/or PE of the order of 4 times for patients under the age of 45 years and 2 times for those over 60 years, although there was no longer an association beyond 75 years of age. The study defined patients with varicose veins as those who had varicose veins or who had undergone some type of procedure for treatment of varicose veins, such as surgery or sclerotherapy. Unfortunately, the time elapsed between the procedure and detection of DVT and/or PE was not reported. Therefore, what is associated with the increased risk of DVT and PE is not just *presence of varicose veins* at the time of the event, but a previous history of *varicose veins and surgical procedures* because of the condition. It is possible that the discrepancy between the odds ratios for the different age groups may be because of the disproportionate rates of invasive procedures to treat varicose veins, since beyond 60 years the proportion of patients treated with surgery or sclerotherapy reduces because of the risks of surgery. This uncertainty is compounded further because the incidence of DVT after surgery for varicose veins is also unknown, although it is presumed to be below 1%. A 2013 study by Testroote and Wittens,^3^ based solely on a systematic search of PubMed, found three prospective studies that tested for DVT during the postoperative period after varicose vein surgery using Doppler ultrasound, but only screened patients with signs and symptoms of DVT. These studies suggested that the incidence of DVT after varicose vein surgery was possibly 5 to 10 times greater than had been presumed.

Müller-Bühl et al.^2^ conducted a retrospective study of 83,143 patients, using an ICD (International Classification of Diseases) codes register maintained by a University Hospital in Germany to correlate presence of varicose veins with DVT and SVT. They demonstrated a relationship, detecting DVT incidence rates of 5.6% vs. 0.9% and SVT rates of 2.1% vs. 0.4%, comparing patients with and without varicose veins. Both comparisons were statistically significant. However, the study’s methodological limitations (retrospective and based on ICD data that could be imprecise) and indications that the study population did not reflect the true situation (since just 3% of the total number of individuals assessed were diagnosed with lower limb varicose veins whereas the prevalence is known to be 10 times greater than this in the general population) prevents us from confirming the accuracy of this association with a high degree of certainty.

When discussing this subject, we cannot omit to mention studies that have suggested that presence of varicose veins is a risk factor for DVT when associated with surgical procedures. A 2016 paper by Tan et al.^4^ presented a systematic review with meta-analysis of the incidence and risk factors of venous thromboembolism in patients in the postoperative period after orthopedic surgeries below the level of the hips, found a correlation between venous thromboembolism and presence of varicose veins, with a relative risk of 3.07 (range: 1.12 to 8.47). In the review, this result was from two different studies with a moderate degree of heterogeneity, from case-control studies, for which the risk of bias was not analyzed, and with a wide confidence interval as a result. In other words, the evidence generated had low and very low levels of certainty.

Zhang et al.^5^ conducted a systematic review with meta-analysis of risk factors for venous thromboembolism, in this case after hip and knee joint replacements, which found a correlation between the presence of varicose veins and symptomatic and asymptomatic venous thromboembolism. In this study, there were also two retrospective studies, with a high degree of heterogeneity and a wide confidence interval, in other words evidence with a very low degree of certainty. Kakkar et al.^6^ conducted a study of the incidence of DVT during the postoperative period after gynecological surgery, finding that presence of varicose veins was an independent factor in development of DVT. Sue-Ling et al.^7^ studied 128 patients in the postoperative period after abdominal surgery and also listed presence of varicose veins as an independent factor in development of DVT. Clayton et al.^8^ studied 124 patients in the postoperative period after gynecological surgery via abdominal and vaginal access, analyzing the incidence of DVT with the aim of identify risk factors and developing a risk score for DVT. The patients studied were not given DVT prophylaxis during the postoperative period. Presence of varicose veins was one of the risk factors related to development of DVT. The studies conducted by Kakkar, Sue-Ling, and Clayton all used radiolabeled anti fibrin antibody for diagnosis of DVT, which is a test that has been abandoned because of low sensitivity, low specificity, and technical difficulties.^9^ When discussing DVT and lower limb varicose veins, we cannot leave out SVT (thrombophlebitis), since it is known that there is a correlation between presence of varicose veins and development of SVT and, in some cases, progression to DVT.

Finally, one option for elucidating this issue would be to conduct a randomized clinical trial recruiting patients with lower limb varicose veins and comparing surgical treatment against conservative treatment with presence of DVT in both groups over the long term as one of the outcomes. This would reveal the incidence of DVT during the postoperative period after varicose vein treatment, the incidence of DVT in a group managed conservatively, and the possible prophylactic effect for DVT and PE of operating on varicose veins and would enable mortality in both groups to be estimated.
