# Supplementary material for: Correlation between presence of lower limb varicose veins and deep venous thrombosis
Source: J Vasc Bras. 2020 Jul 31;19:e20200081. doi: 10.1590/1677-5449.200081 (PMC8202180; doi:10.1590/1677-5449.200081)
Supplement: Translation

# Translation: Correlação entre a presença de varizes de membros inferiores e trombose venosa profunda

**Authors:** Daniel Guimarães Cacione, Frederico do Carmo Novaes, José Carlos Costa Baptista Silva

**Affiliations:** 1 Universidade Federal de São Paulo – UNIFESP, Escola Paulista de Medicina – EPM, São Paulo, SP, Brasil.

A associação entre a presença de varizes de membros inferiores (MMII) e a ocorrência de trombose venosa profunda (TVP) é um tema controverso na literatura. A questão sobreveio a partir de estudos que buscavam elencar os fatores de risco para TVP, principalmente em pós-operatórios, para instaurar a profilaxia para TVP e, paralelamente, a partir da pesquisa acerca da trombose venosa superficial (TVS), também conhecida como tromboflebite superficial, uma vez que pode evoluir ou estar relacionada com a TVP. A controvérsia surge porque os estudos que pesquisam a associação apresentam inúmeros problemas que não permitem um grau razoável de certeza. Em teoria, o desenho de estudo mais apropriado para a pesquisa da associação de um fator de risco (ex., varizes de MMII) e uma doença (ex., TVP) é a coorte. No entanto, esse desenho não foi utilizado para provar a associação. Com um grau de confiabilidade um pouco menor, temos os estudos de caso controle, em que partimos dos pacientes com a doença e pesquisamos os fatores de risco para o seu desenvolvimento. É esse formato de estudo que abriga a maioria dos trabalhos acerca do tema.

Dois estudos de caso controle populacionais pesquisam a associação de varizes e TVP: Heit et al.^1^ e Müller-Bühl et al.^2^, publicados em 2002 e 2012, respectivamente. No estudo de Heit et al.^1^, um total de 1.250 pacientes, em acompanhamento de 25 anos, foram separados em dois grupos: 625 com TVP e embolia pulmonar (EP) foram comparados com 625 sem TVP ou EP no grupo controle. Após realização de análise logística multivariada, encontrou-se uma associação da presença de varizes e o desenvolvimento de TVP e/ou EP da ordem de 4 vezes para pacientes com menos de 45 anos e de 2 vezes para maiores de 60 anos, não havendo associação acima de 75 anos. Nesse estudo, foram considerados portadores de varizes aqueles pacientes que apresentavam veias varicosas ou que haviam realizado algum procedimento para o tratamento das varizes, tais como cirurgia e escleroterapia. Infelizmente, o tempo decorrido entre o procedimento e a detecção da TVP e EP não foi divulgado. Assim, o que se associa ao risco aumentado de TVP e EP é não só a *presença de varizes* no momento do evento, mas a história pregressa de *varizes e procedimentos cirúrgicos* em decorrência da doença. Talvez a discrepância entre a razão de chances nas diversas faixas etárias possa ser em decorrência da desproporção de procedimentos invasivos para o tratamento das varizes, uma vez que acima de 60 anos diminui-se a proporção de doentes tratados com cirurgia ou escleroterapia por conta do risco cirúrgico. A incerteza se agrava ainda mais uma vez que a incidência de TVP após a cirurgia de varizes também é desconhecida, presumindo-se que esteja abaixo de 1%. O trabalho de Testroote and Wittens^3^, de 2013, em pesquisa sistemática somente na PubMed, encontrou três trabalhos prospectivos em que foi feita a pesquisa de TVP em pós-operatório de cirurgia de varizes através do ultrassom Doppler somente para doentes com sinais e sintomas de TVP. Esses estudos sugerem que a possibilidade da incidência de TVP em pós-operatório de varizes pode ser de 5 a 10 vezes maior que a presumida.

No trabalho de Müller- Bühl et al.^2^, foi realizado um estudo retrospectivo abrangendo 83.143 pacientes, utilizando um banco de CIDs (Código Internacional de Doenças) de um hospital universitário na Alemanha para correlacionar a presença de varizes com TVP e TVS. Os autores demonstram a relação, detectando uma incidência para TVP 5,6% *versus* 0,9% e, para TVS, 2,1% versus 0,4%, comparando-se pacientes com e sem varizes, sendo ambas as comparações estatisticamente significantes. No entanto, as limitações metodológicas do estudo (retrospectivo, baseado em informações de CID que podem ser imprecisas) e o indício de que a população estudada não reflita a realidade — uma vez que apenas 3% do total de indivíduos avaliados foram diagnosticados com varizes de MMII e tendo em vista que a prevalência é sabidamente 10 vezes maior na população geral — não nos permitem afirmar com alto índice de certeza a veracidade dessa associação.

Nesta discussão, não podemos deixar de citar os estudos que sugerem a presença de varizes como fator de risco para TVP quando associadas a procedimentos cirúrgicos. O trabalho de Tan et al.^4^, de 2016, uma revisão sistemática com metanálise sobre a incidência e fatores de risco para tromboembolismo venoso em pacientes em pós operatório ortopédico abaixo do nível do quadril, encontrou uma correlação entre tromboembolismo venoso e presença de varizes, com valor de risco relativo de 3,07 (variando de 1,12 até 8,47). Nessa revisão, esse resultado veio de dois estudos, com moderada heterogeneidade entre eles, de estudos caso controle, não se avaliando o risco de viés desses estudos, além do amplo intervalo de confiança resultante. Ou seja, gerou-se uma evidência com baixo a muito baixo grau de certeza.

O estudo de Zhang et al.^5^, também uma revisão sistemática com metanálise sobre fatores de risco para tromboembolismo venoso após artroplastia de quadril e joelho, mostrou uma correlação entre a presença de varizes e tromboembolismo venoso sintomático e assintomático. Aqui, novamente, temos dois estudos retrospectivos, com alta heterogeneidade e intervalo de confiança largo — ou seja, evidência com baixo a muito baixo grau de certeza. No estudo de Kakkar et al.^6^ sobre a incidência de TVP em pós-operatório ginecológico, a presença de varizes foi relacionada como fator independente para desenvolvimento de TVP. O estudo de Sue-Ling et al.^7^, com 128 pacientes em pós-operatório abdominal, relacionou a presença de varizes como fator independente para desenvolvimento de TVP. No estudo de Clayton et al.^8^, envolvendo 124 pacientes em pós-operatório ginecológico por via abdominal e vaginal, foram estudados com relação ao aparecimento de TVP, em vistas a identificar fatores de risco e confeccionar um escore de risco para TVP. Os pacientes estudados não utilizaram profilaxia para TVP no pós-operatório. A presença de veias varicosas foi um dos fatores de risco relacionados ao desenvolvimento de TVP. Os trabalhos de Kakkar, Sue e Clayton utilizaram como diagnóstico de TVP o uso de anticorpo antifibrinogênio radioativo, exame que foi abandonado por conta da baixa sensibilidade, baixa especificidade e dificuldades na sua realização^9^. Na discussão de TVP e varizes de MMII, não podemos deixar de falar sobre a TVS (tromboflebite), uma vez que é sabida a correlação entre a presença de varizes e o desenvolvimento da TVS e, em alguns casos, a progressão para TVP.

Por fim, uma possibilidade para esclarecermos a questão seria a realização de um ensaio clínico randomizado envolvendo pacientes com varizes de MMII, comparando o tratamento cirúrgico *versus* conservador e tendo como desfechos a presença de TVP em ambos os grupos a longo prazo. Assim, poderíamos conhecer a incidência de TVP no pós-operatório de varizes, a incidência de TVP no grupo com tratamento conservador e o possível efeito profilático da operação de varizes para a TVP e EP, inclusive com estimativa da mortalidade nos dois grupos.
